# Finishing General and Adult Psychiatric Training During COVID-19 and Getting Prepared to Become a Post-Pandemic Psychiatrist in Central Europe

**DOI:** 10.1192/j.eurpsy.2022.196

**Published:** 2022-09-01

**Authors:** N. Zaja, A. Seker

**Affiliations:** 1 University Psychiatric Hospital Vrapce, Department For Biological Psychiatry And Psychogeriatrics, Zagreb, Croatia; 2 Cambridge and Peterborough NHS Foundation Trust, Child/adolescent Psychiatry, Cambridge, United Kingdom

## Abstract

The ongoing pandemic has brought many changes to the lives of almost all people around the world. Medical professionals have also experienced significant changes at their workplace, with many of them engaged in treating COVID-19 patients instead of working in a field of medicine they have trained to work in. Trainees of all medical specialities have often been the first ones to join the effort in combating the pandemic and psychiatric trainees were no exception. The pandemic has brought their training to a halt and the majority of psychiatric educational activities have initially been postponed or canceled, with clinical rotations between different institutions almost non-existent. In a search for more space to treat COVID-19 patients, psychiatric wards were often the first ones to be repurposed. Epidemiological measures have lowered the number of patients a trainee could see each day and have also made clinical supervision less available. However, the introduction of online communication platforms as a new standard of interaction has helped mitigate many of the issues and enabled resuming most of the educational activities. Since trainees were usually more experienced in online communication than senior doctors, their skills were very valuable in establishing telepsychiatric services. Even though the pandemic has created many obstacles in psychiatric training, it has also proved that training is very much about learning and implementing new skills, as well as adapting to new circumstances.

**Disclosure:**

No significant relationships.

